# One- and Two-Band Sensors and Algorithms to Derive *a*_CDOM_(440) from Global Above- and In-Water Optical Observations

**DOI:** 10.3390/s21165384

**Published:** 2021-08-09

**Authors:** Stanford B. Hooker, Henry F. Houskeeper, Randall N. Lind, Koji Suzuki

**Affiliations:** 1 NASA Goddard Space Flight Center, Greenbelt, MD 20771, USA; 2 Department of Geography, University of California, Los Angeles, CA 90095, USA; hhouskee@g.ucla.edu; 3 Biospherical Instruments Inc., San Diego, CA 92110, USA; randy@biospherical.com; 4 Faculty of Environmental Earth Science, Hokkaido University, Sapporo 060-0810, Japan; kojis@ees.hokudai.ac.jp

**Keywords:** ocean color, global (oceanic, coastal, and inland) waters, end members, radiometers, PAR, hybridnamic, autonomous (AUV, USV, UAV, and float) platforms, remote sensing

## Abstract

The colored (or chromophoric, depending on the literature) dissolved organic matter (CDOM) spectral absorption coefficient, $a_{\mathrm{CDOM}}\left(\lambda \right)$, is a variable of global interest that has broad application in the study of biogeochemical processes. Within the funding for scientific research, there is an overarching trend towards increasing the scale of observations both temporally and spatially, while simultaneously reducing the cost per sample, driving a systemic shift towards autonomous sensors and observations. Legacy $a_{\mathrm{CDOM}}\left(\lambda \right)$ measurement techniques can be cost-prohibitive and do not lend themselves toward autonomous systems. Spectrally rich datasets carefully collected with advanced optical systems in diverse locations that span a global range of water bodies, in conjunction with appropriate quality assurance and processing, allow for the analysis of methods and algorithms to estimate $a_{\mathrm{CDOM}}\left(440\right)$ from spectrally constrained one- and two-band subsets of the data. The resulting algorithms were evaluated with respect to established fit-for-purpose criteria as well as quality assured archival data. Existing and proposed optical sensors capable of exploiting the algorithms and intended for autonomous platforms are identified and discussed. One-band in-water algorithms and two-band above-water algorithms showed the most promise for practical use (accuracy of 3.0% and 6.5%, respectively), with the latter demonstrated for an airborne dataset.

## 1. Introduction

Autonomous oceanographic platforms, e.g., an autonomous underwater vehicle (AUV) or an unmanned surface vehicle (USV), offer sampling benefits regardless of the size of the water mass, because they remove the cost of supporting a human operator. When the water body is very large or if a long time series is desired, the advantages are more significant, because of the cost of deploying the requisite research team(s) for an extended time period. If the sampling site is difficult to reach or dangerous for humans, e.g., polar water masses, researcher deployment costs increase further. Although the benefits of autonomous sampling include other factors, the trajectory of increasing costs to achieve a global sampling benefit is an underlying motivation for the study presented herein.

Of course, autonomous systems are also expensive and, in general, the more capable they are, the higher the cost. For example, a longer duration system requires a more sophisticated power system, which frequently translates into a larger or complicated rechargeable battery circuit. To minimize the cost per sample ratio, lowering the amount of power consumed to obtain a useful data product is a key design objective. This may also be expressed in terms of weight, because for many autonomous platforms—for example, an unmanned aerial vehicle (UAV)—weight can be a significant cost driver.

For many design teams, the cost-benefit and cost-sample ratios are principal components for an overall risk-reward paradigm. The discussion above is purposefully brief and does not capture all the factors influencing the ultimate risk-reward ratio that determines whether or not a particular autonomous sampling system is used. In general, however, the more complicated the device and the more remote the operation, the greater the risk and likely the greater the reward. An example of the ultimate expression of this dichotomy is a spaceborne sensor.

This study is not based on just one aspect of the risk-reward spectrum, e.g., complicated and costly satellites. Instead, the perspective adopted here is to minimize risk and maximize reward with an approach that is applicable to a wide diversity of above- and in-water autonomous systems capable of sampling inland, coastal, and oceanic waters, i.e., capable of contributing to a diversity of scales, including global research. The global perspective requires a data product that is applicable to all scales, which means sensor systems typically deployed at those scales must be able to produce the data product with sufficient accuracy (i.e., low uncertainty) to be scientifically useful.

The selected data product is the colored dissolved organic matter (CDOM) spectral absorption coefficient, $a_{\mathrm{CDOM}}\left(\lambda \right)$, where λ is wavelength. The temporal and spatial distribution of $a_{\mathrm{CDOM}}\left(\lambda \right)$ is widely used to investigate terrestrial and aquatic biogeochemical processes at multiple scales [1,2].

CDOM is remotely sensed, impacts remote sensing algorithms, and can vary on short time scales [3]. Developing algorithms for the in situ estimation of CDOM that are globally applicable, and optimizing sensors to exploit these advances that are globally capable, both in terms of radiometric performance and viability for network-scale observations, is of interest to the scientific community. The selection of $a_{\mathrm{CDOM}}\left(440\right)$ as the parameter of interest is motivated by the relationships between CDOM and the solar illumination of aquatic ecosystems as documented by many researchers, e.g., [4,5,6,7].

The global perspective for applying an algorithm based on near-surface observations is first considered based on the dynamic range of $a_{\mathrm{CDOM}}\left(440\right)$, which spans more than three decades in this study, i.e., 0.001–2.146 m${}^{-1}$, and includes similar representation from oceanic, coastal, and inland waters. As a log-normal variable, unsampled waters wherein $a_{\mathrm{CDOM}}\left(440\right)$ exceeds 2.146 m${}^{-1}$ are unlikely to significantly expand the range in $a_{\mathrm{CDOM}}\left(440\right)$ when considered in log space. Furthermore, algorithms derived using a narrower $a_{\mathrm{CDOM}}\left(440\right)$ range in Houskeeper [8] produced similar algorithmic coefficients as those derived using an expanded $a_{\mathrm{CDOM}}\left(440\right)$ range in Houskeeper et al. [9], due to the log-space linearity of the relationship between $a_{\mathrm{CDOM}}\left(440\right)$ and ratios of optical observations at UV and NIR wavebands. A perspective that accounts for observations uniformly spread across oceanic, coastal, and inland waters, while spanning more than three decades of the log-normal variable, is considered herein as global.

The global perspective for applying an $a_{\mathrm{CDOM}}\left(440\right)$ algorithm derived from near-surface observations is also considered based on aquatic surface area. If the surface area of the oceans, marginal seas, and the 30 largest inland water bodies [10], for which $a_{\mathrm{CDOM}}\left(440\right)$ is on average less than 1 m${}^{-1}$, are removed from the global aquatic surface area, less than 1% of the surface area remains. Although applicability to more than 99% of the aquatic surface area can arguably be considered global, the surface area of rivers and streams [11] —which have residence times generally preventing extreme concentrations—further reduces the percentage of the global aquatic surface area exceeding the three decades of dynamic range considered herein.

The most direct observation of $a_{\mathrm{CDOM}}\left(440\right)$ requires a laboratory water sample analysis and is not well suited for autonomous sampling. Recent advances with spectral end-member analysis (EMA) by Hooker et al. [12], wherein optical measurements obtained at discrete wavebands (typically 10 nm wide) are inverted to derive $a_{\mathrm{CDOM}}\left(440\right)$, are compatible with autonomous sampling because only two wavelengths are used, i.e., usually the shortest and longest. More importantly, Hooker et al. [13] showed that the two-channel (i.e., band-ratio) EMA approach spans a global distribution in $a_{\mathrm{CDOM}}\left(440\right)$ derived from in-water observations of the diffuse attenuation coefficient, $K_{d}\left(\lambda \right)$. Houskeeper et al. [9] extended that work by demonstrating a global relationship between $a_{\mathrm{CDOM}}\left(440\right)$ and the normalized water-leaving radiance, ${\left[L_{W}\left(\lambda \right)\right]}_{N}$, which can be derived from above- and in-water optical observations (discussed below).

Some of the above- and in-water optical observations used in Houskeeper et al. [9] and Hooker et al. [13] were obtained from semi-autonomous platforms manufactured by Biospherical Instruments Inc. (San Diego, CA, USA), respectively: (a) the Compact-Airborne Environmental Radiometers for Oceanography (C-AERO) above-water instrument suite, which operates autonomously on a manned aircraft [14]; and (b) the Compact-Optical Profiling System (C-OPS) in-water instrument suite with Compact-Propulsion Option for Profiling Systems (C-PrOPS) accessory [14], which has two small digitally controlled thrusters to allow profiling independent of the primary platform, e.g., a research vessel or shoreline, or from a USV [15]. Both of these instrument systems use the Biospherical Instruments Inc. (BSI) microradiometer [16], an independent networkable miniaturized radiometer (Section 2), as the core sensor technology. This study extends the use of the latter for new autonomous applications based on smaller instruments as described below.

These sampling alternatives produced data of equal quality in comparison to fully manned systems, but in both cases the optical systems were complex. For example, all of them used three radiometers equipped with 19 channels, which means they are not easily exploited for long-duration deployments, because of bio-fouling and power concerns, or for a UAV, because of size and power constraints. To ensure cost efficiency, a single design applicable to both above- and in-water autonomous platforms is appealing as is a very small instrument to reduce weight and power requirements. The smallest and lowest power instrument would be a one-band radiometer, but at present the simplest, common $a_{\mathrm{CDOM}}\left(440\right)$ algorithms use two or more wavelengths, e.g., [17]. It would also be desirable if the instrument has a dynamic range satisfying the global requirements for water bodies.

As described by Hooker et al. [13] and Houskeeper et al. [9], $a_{\mathrm{CDOM}}\left(440\right)$ can be inverted from $K_{d}\left(\lambda \right)$ and ${\left[L_{W}\left(\lambda \right)\right]}_{N}$ observations using two or more wavelengths, i.e., including multispectral techniques. The advantages and disadvantages of $K_{d}$ versus ${\left[L_{W}\left(\lambda \right)\right]}_{N}$ are as follows: (a) $K_{d}\left(\lambda \right)$ can be obtained accurately from an uncalibrated—but stable—instrument, whereas ${\left[L_{W}\left(\lambda \right)\right]}_{N}$ cannot [12]; (b) ${\left[L_{W}\left(\lambda \right)\right]}_{N}$ can be obtained remotely, whereas inversion schemes to obtain $K_{d}\left(\lambda \right)$ remotely are spectrally incomplete and introduce undesirable inaccuracies, e.g., [18]; (c) $K_{d}\left(\lambda \right)$ does not require a self-shading correction, but ${\left[L_{W}\left(\lambda \right)\right]}_{N}$ does; and (d) for long-term deployments (e.g., on unmanned platforms), techniques to mitigate aperture biofouling are more straightforward for ${\left[L_{W}\left(\lambda \right)\right]}_{N}$, as opposed to $K_{d}\left(\lambda \right)$, data products [19]. For the latter, UV irradiation or stowage at great water depth when not sampling are potentially useful fouling mitigators.

The offsetting pros and cons of using $K_{d}\left(\lambda \right)$ and ${\left[L_{W}\left(\lambda \right)\right]}_{N}$ for a global single-band $a_{\mathrm{CDOM}}\left(440\right)$ algorithm mean both are potentially attractive, with the former more attractive for an in-water platform and the latter for an above-water platform. Consequently, the objectives of this study are as follows: (a) establish the efficacy of one-band global algorithms to derive $a_{\mathrm{CDOM}}\left(440\right)$ from above- and in-water platforms obtained from highly accurate one-band microradiometer measurements from which ${\left[L_{W}\left(\lambda \right)\right]}_{N}$ or $K_{d}\left(\lambda \right)$, respectively, are derived; (b) assess one-band algorithms versus established [9,13] two-band algorithms; and (c) provide notional concepts for the one- and two-channel instruments to be integrated onto existing autonomous platforms.

## 2. Materials and Methods

The dataset used for the study herein is a subset of the [13] open ocean, coastal zone, and inland waters sampling area. The subset is based on selecting stations classified as representing conservative and near-conservative water masses. A water mass is considered conservative if the inflow and outflow of properties constrain the range in the gradient of a constituent [13]. A near-conservative water body is a slightly modified portion of a larger parent water body that is conservative. For example, a water mass within line-of-sight proximity of the marginal ice zone (MIZ), but far from it such that MIZ meltwater has only a small influence on water properties associated with the surrounding parent water body [13], is considered near-conservative. The influence of uncertainty sources that are absent from conservative or near-conservative water masses is provided in Hooker et al. [13,20].

The geographical area for the dataset is as follows: Japan, the western North Pacific Ocean (e.g., the Kuroshio and Oyashio Currents), the central North Pacific Ocean, the Bering Sea, the Chukchi Sea, the Beaufort Sea, the western U.S. (i.e., California, Oregon, Washington, Nevada, Utah, and Idaho), Hawaii, and Puerto Rico. Data collection spanned 29 April 2013 to 13 September 2017. The inclusion of near-conservative samples expands the [13] dataset from 613 exclusively conservative samples, as used in Houskeeper et al. [9], to 789 samples. The expansion allows a cross-validation analysis, wherein 80% of the total dataset is randomly selected for algorithm fitting and compared to the remaining 20%. Repetitive random selection and analysis allows for a statistical description of algorithm efficacy (described below).

**Figure 1:**
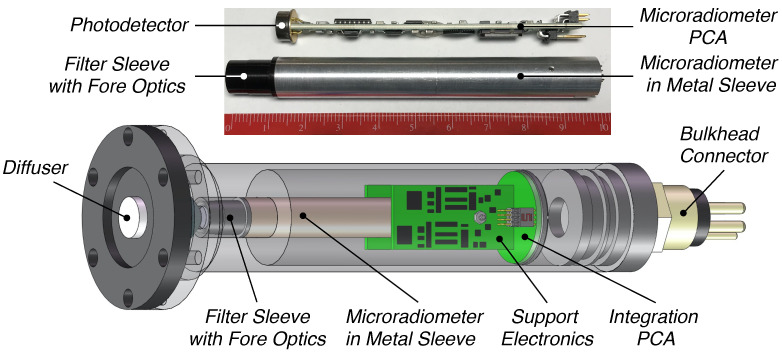
The two-sided microradiometer PCA with photodetector positioned (top) above a metal-sleeved unit with black front-end optics (red ruler to 10 cm). A single-channel (PAR or fixed wavelength) irradiance sensor (bottom and not to the same scale) optimized for autonomous in-water platforms (e.g., an AUV) with 2.0 in (5.1 cm) diameter diffuser (cosine collector) attached to a 1.2 in (3.0 cm) diameter housing that is 7.2 in (18.3 cm) long. The total weight is 0.6 lb (0.3 kg).

Measurements of the apparent optical properties (AOPs) of water masses were obtained with instruments built as a cluster of 19 microradiometers, wherein a microradiometer is a fully functional instrument built with a single printed circuit assembly (PCA). A microradiometer is explained in detail by Morrow et al. [16], so only a brief description is as follows: (a) the device consists of a photodetector, three-gain preamplifier, 24 bit analog-to-digital converter (ADC), microprocessor, and an addressable digital port; (b) the sampling rate is typically 15 Hz, with the newest generation capable of 30 Hz sampling; (c) the linear dynamic range spans 10 decades, i.e., it can view the sea and Sun without saturating and is sensitive enough to view the Moon with no loss in precision or accuracy (most radiometers have 5–6 decades of dynamic range and are not as capable); and (d) it is a fully networkable sensor on one small, thin, conformal-coated PCA sleeved inside a metal cylinder for mechanical support and electromagnetic shielding. The microradiometer sensor building block, although originally deployed in clusters, lends itself to single-channel sensors (Figure 1).

The microradiometer PCA is machine assembled, which substantially improves quality over handmade legacy devices. The support electronics include a precision direct current (DC) power supply and may contain an aggregator, which creates a composite device, if the instrument design includes a cluster of microradiometers or optional ancillary sensors, e.g., water temperature and pressure. Although the inclusion of ancillary sensors is easily supported by the microradiometer architecture, the perspective of this study is that ancillary sensors are present on the integration platform hosting the optical sensor(s). Consequently, the optical sensors presented here omit ancillary sensors in favor of an optimization driven by the reduction in mass and power consumption. Demonstrated life cycles for instruments built with microradiometers presently exceed 15 years with a failure rate well below 1%. The 10 decades of dynamic range and 15–30 Hz high-speed sampling allow above- and in-water light contamination effects to be properly dealt with, as follows (respectively): surface light perturbations due to oblique wave facets that reflect sunlight can be discretized and properly removed [9], and the central tendency of non-Gaussian brightening from wave focusing can be properly determined with minimal bias [13].

Relative to single-channel legacy measurement capabilities (e.g., the BSI QCP-2150), the mass of the Figure 1 instrument has been reduced (nominally 50%) to minimize impact on the energy budget for the overall sampling system. The mass reduction is notable, because while the irradiance sensor does not suffer from self-shading concerns, its requisite positioning at the top of the integrated platform means its mass must be at least partially driven above the air–water interface during autonomous profiling, e.g., for satellite communications. The depth rating is restricted to 2000m as part of the mass reduction, but is consistent with the common float populations servicing the global sampling community, e.g., profiling floats. The integration of the microradiometer to a single-channel application may extend the noise-equivalent irradiance (NEI) of in-water PAR observations by up to four decades of dynamic range compared to legacy technologies without a multistage preamplifier.

**Figure 2:**
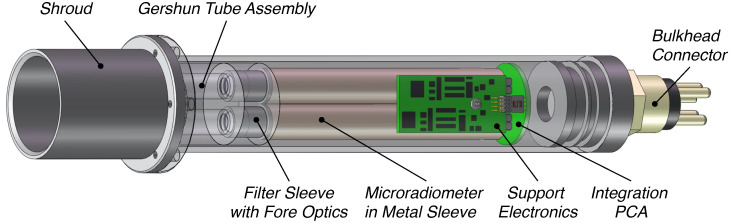
A two-channel radiance sensor optimized for autonomous above-water platforms (e.g., a UAV) with a 1.3 in (3.4 cm) diameter and 1.5 in (3.8 cm) long shroud attached to a 1.2 in (3.0 cm) diameter housing that is 7.5 in (19.1 cm) long. The total weight is 0.5 lb (0.2 kg).

Utilizing the same nominal form factor as the single-channel irradiance sensor, a two-channel radiance embodiment for above-water sampling is presented in Figure 2. The second spectral channel permits band-ratio algorithms to be implemented in environmental scenarios where the results from single-channel algorithms may be diminished (Section 4). The Gershun tubes limit the half-angle field of view (FOV) to 5${}^{\circ }$, and the shroud mitigates environmental contamination and prevents scattering from off-angle incident light at long wavelengths, e.g., the short-wavelength infrared (SWIR). The shroud is removable (if not needed), which facilitates easy cleaning of the optical aperture and customization for unique mounting scenarios. Although anticipated for above-water radiometry for deriving ${\left[L_{W}\left(\lambda \right)\right]}_{N}$, the sensor is equally applicable for in-water use and has a depth rating of 2000 m. Variations of the sensor with greater depth capability are easily fabricated with the tradeoff of increased mass. Although sensitive to self-shading effects and solar geometry requirements, an in-water radiance sensor does have the following benefits: (a) immersion factors are easily computed; (b) mass is less critical to the power budget of an autonomous profiler, because the sensor is usually not driven above the air–water interface, and the aperture is typically pointed downwards, thereby mitigating bio-fouling or sediment deposition.

### 2.1. C-OPS In-Water Optical Observations

In-water AOPs were measured with a handheld, free-falling C-OPS instrument suite [16]. For almost all data acquisition (97%), C-OPS was deployed with the C-PrOPS accessory, which has two small thrusters [14] that propel and steer (using differential thrust) the profiler backplane into the sampling location. Thrusters also improve the quality of data products as a result of a multitude of sampling advantages that cannot be similarly achieved without their use [14], so only brief descriptions of important aspects are presented here, as follows:

1. The backplane is stabilized prior to profiling, which increases the amount of data obtained within planar orientation limits (only in-water light observations level to within 5${}^{\circ }$ are used to derive data products);
2. Combined with C-OPS hydrobaric buoyancy (i.e., varying inversely with depth) and a 15 Hz data rate, low thrust levels reduce the velocity of profiler descent, thereby improving the vertical sampling resolution (VSR), i.e., the extrapolation layer thickness (set by $z_{1}$ and $z_{2}$) divided by the number of planar samples obtained;
3. Differential thrust applied prior to profiling aligns the backplane with respect to the solar principal plane to minimize self-shading effects;
4. Thrusters keep the profiler at the surface before profiling commences with the pressure transducer measuring atmospheric pressure, so each profile can be individually pressure tared;
5. In non-navigable water masses (e.g., a shallow river or a lake closed to small boats to prevent the spread of invasive species), the profiler can be launched from the shoreline; and
6. At the bottom of the profile (typically the 1% light level, but at least the 10% light level to remove perturbations from bottom reflection), thruster power prevents depth overshoot and the likelihood of a bottom impact, plus the subsequent rapid return of the profiler to the surface decreases the time lapse between profiles, thereby minimizing environmental variability effects and cast-to-cast differences in data products (especially in heterogenous water masses).

The three C-OPS radiometers simultaneously measure the in-water downward irradiance ($E_{d}$) and upwelling radiance ($L_{u}$), plus the above-water global solar irradiance ($E_{s}$). The radiometers have 19 identical wavebands with 10 nm bandwidths spanning the ultraviolet (UV), visible (VIS), and near-infrared (NIR) domains, i.e., 313–875 nm. The 313 nm waveband was added as part of an instrumentation upgrade after field sampling commenced, so 313 nm is present for 55.4% of the data and all other wavelengths used herein are always present.

The ability to derive in-water data products at any wavelength depends on the optical characteristics of the water mass and strict adherence to the NASA Ocean Optics Protocols [21,22,23], hereafter The Protocols, encompassing the requirements for instrument specifications and characterization, illumination and environmental conditions in the field, plus data acquisition, and data processing. The latter two are significantly important because they must properly implement a substantive inventory of corrections based on laboratory and field characterizations as applicable. A comprehensive inventory of applicable corrections are presented by Hooker et al. [15], so only a brief summary is presented, as follows: (a) non-real-time serial communications; (b) individual instrument characterizations and calibrations; (c) gain stage transitions and dark current characterizations in the field (manual capping of the apertures); (d) illumination geometry and normalization; (e) aperture tilting and planar offsets; (f) temperature-induced and responsivity nonlinearities; (g) transducer hysteresis and pressure tares, plus (h) backplane and instrument self-shading.

### 2.2. In-Water C-OPS Irradiance Data Products

In-water C-OPS data products were derived by selecting upper ($z=z_{1}$) and lower ($z=z_{2}$) depths to define an extrapolation interval within a homogenous near-surface layer. The resulting derived irradiance data products were evaluated by comparing $E_{s}\left(\lambda \right)$ transmitted through the interface to null depth ($z=0^{-}$ m) and $E_{d}\left(\lambda \right)$ extrapolated to null depth. If the two products did not agree to within the calibration uncertainty (nominally 3%), $z_{1}$ and $z_{2}$ were redetermined—while keeping both within the shallowest homogeneous layer [13]. This process was repeated on a profile-by-profile basis [12], with all extrapolation intervals kept as close to the water surface as possible. The average $z_{2}$ value for the dataset was 0.7 m (i.e., all data products were derived in the top 1 m of the water column). The average planar tilt and descent velocity within the extrapolation interval were 1.4${}^{\circ }$ and 0.1 m s^−1^, respectively, and the average VSR was 0.6 cm, but for very shallow or turbid waters, the average VSR was 0.9 mm.

The $K_{d}\left(\lambda \right)$ data products are obtained from the individual $E_{d}\left(\lambda \right)$ regressions. Following The Protocols, the latter includes more than 5 wavelengths spanning the VIS domain (approximately 13 plus 2 VIS neighbors at 395 nm and 710 nm) and can be used to accurately derive photosynthetically available radiation (PAR). The computation of PAR requires the following: (a) converting irradiance (energy) to quanta, (b) weighting the contributions based on the separation between center wavelengths, and (c) integrating across the wavelengths to calculate PAR. A spectrally *bulk* measure of attenuation is derived from the PAR estimates as a function of depth following the same regression procedures used to derive $K_{d}\left(\lambda \right)$ and is denoted $K_{d}\left(\mathrm{PAR}\right)$ to emphasize the derivation of PAR from $E_{d}\left(\lambda \right)$.

The computation of PAR from discrete bands allows ultimate control over the weighting of the bands within the VIS domain. The use of broadband filters to accomplish the same observation using a single PAR channel (Figure 1) tends to result in more variability through the passband, e.g., as a result of turn-on and cutoff wavelength differences. Secondary filter combinations can be used to tune each overall filter response to be within certain bounds, but the consistency and stringency is less than what can be achieved with discrete spectral bands.

Assuming $K_{\mathrm{PAR}}$ represents the attenuation of PAR measured with a one-band PAR instrument, a comparison of $K_{d}\left(\mathrm{PAR}\right)$ versus $K_{\mathrm{PAR}}$ for C-OPS instruments containing a separate PAR channel and at least 13 VIS channels, shows agreement to within the uncertainty of calibration plus a nominal contribution from environmental variance (principally wave-focusing effects), i.e., less than 3.5% [14]. These differences are not considered significant for the results presented herein, because the algorithmic approach involving PAR is based on the attenuation of PAR in the water column rather than the absolute value of PAR.

### 2.3. In-Water C-OPS Radiance Data Products

The C-OPS $L_{u}\left(\lambda \right)$ regressions within the extrapolation interval defined by $z_{2}$ and $z_{1}$ yield the individual extrapolations to null depth, $L_{u}(0^{-},\lambda )$ from which the in-water derivation of the spectral water-leaving radiance, ${\tilde{L}}_{W}\left(\lambda \right)$, is obtained directly, as follows (with geometrical terms removed for brevity, but it is important to recall $L_{u}(\lambda )$ is observed from a nadir-viewing radiometer):

(1) $$\begin{array}{c} {\tilde{L}}_{W}\left(\lambda \right)\;=\;0.54\;L_{u}(0^{-},\lambda ), \end{array}$$

where the constant 0.54 accurately accounts for the partial reflection and transmission of the upwelled radiance through the water surface (Mobley 1999). A bulk or broadband equivalent for PAR, but based on ${\left[L_{W}\left(\lambda \right)\right]}_{N}$, is not proposed or used herein.

To account for dependencies on the solar flux, which is a function of atmospheric conditions and time of day, the ${\tilde{L}}_{W}$ term is normalized by $E_{s}$ measured during the time interval corresponding to $z_{1}$ and $z_{2}$:

(2) $$R_{\mathrm{rs}}\left(\lambda \right)\;=\;\frac{{\tilde{L}}_{W}\left(\lambda \right)}{E_{s}\left(\lambda \right)},$$

where $R_{\mathrm{rs}}$ is the remote sensing reflectance. An additional refinement includes the bidirectional nature of the upwelled radiance field, which is to a first approximation considered as the hypothetical water-leaving radiance that would be measured in the absence of any atmospheric loss with a zenith Sun at the mean Earth–Sun distance [24,25]. The latter is accomplished by adjusting $R_{\mathrm{rs}}\left(\lambda \right)$ with the time-dependent mean extraterrestrial solar irradiance, $F_{0}$ (ignoring all dependencies except wavelength for brevity):

(3) $${\left[L_{W}\left(\lambda \right)\right]}_{N}\;=\;F_{0}\left(\lambda \right)\;R_{\mathrm{rs}}\left(\lambda \right),$$

where $F_{0}\left(\lambda \right)$ is obtained from look-up tables [26] by applying a sequential day of the year correction. An additional correction for a so-called *exact* normalized water-leaving radiance is required for satellite and sea-truth matchups, as explained in The Protocols, but that level of completeness is not needed here (discussed in more detail below). Although only a few wavelengths are presented herein, the dataset includes data products for all 19 wavelengths acquired in all water masses. When considering the $K_{d}\left(\lambda \right)$ or ${\left[L_{W}\left(\lambda \right)\right]}_{N}$ data products herein, an important distinction is that in-water $E_{d}\left(z,\lambda \right)$ data used to derive $K_{d}\left(\lambda \right)$ was obtained as shallow as z=3.0 cm, whereas the in-water $L_{u}\left(z,\lambda \right)$ data used to derive ${\left[L_{W}\left(\lambda \right)\right]}_{N}$ was never shallower than z=0.3 m (due to the length of the downward-pointing $L_{u}$ radiometer).

### 2.4. Above-Water C-AERO Optical Observations

The C-AERO airborne instrument suite uses three above-water radiometers that are functionally identical to the C-OPS radiometers but are configured differently to simultaneously measure $E_{s}$ plus the total radiance measured at the sea surface ($L_{T}$) and the indirect sky radiance ($L_{i}$). The latter two radiometers are fitted with shrouds to minimize long-wavelength scattering at the glass aperture [15]. The instruments have 16 wavelengths that match the C-OPS UV–NIR domains plus 3 in the SWIR domain. In addition, C-AERO radiometers sampled at both 15 and 30 Hz, whereas C-OPS only sampled at 15 Hz. The $L_{T}$ observations are obtained at a specified angle with respect to nadir (typically 40${}^{\circ }$), and $L_{i}$ is measured in the same plane, but at a complementary zenith angle as $L_{T}$. The aircraft flies into or out of the principal solar plane with the radiance instruments pointed abeam, i.e., $L_{T}$ and $L_{i}$ observations are obtained perpendicular to the principal plane with a planar stability as The Protocols require.

The airborne radiance radiometers have a narrow field of view (2.5${}^{\circ }$ full view angle) and can also be used for sun photometry [27]. The first six items in the inventory of corrections described for in-water observations and based on laboratory and field characterizations (Section 2.1) were also applied for above-water observations, with the following clarifications: (a) a spectrally-dependent synthetic or predictive dark current method—based on an operational range of environmental and instrument-specific parameters characterized in the laboratory—ensures an accurate removal of the dark current bias during flight when the instrument apertures are not accessible [28]; (b) flight data were acquired at the lowest safe altitude (LSA), typically about 100 ft (30.5 m), making elevation dependencies (e.g., atmospheric correction) negligible; and (c) self-shading corrections are not required, because the $L_{T}$ radiometer is pointed perpendicular to the aircraft heading into or out of the principal plane and at a 40${}^{\circ }$ nadir angle, so the surface spot is significantly far from the aircraft shadow, which is in the principal plane.

### 2.5. Airborne Data Products

The determination of the water-leaving radiance from airborne observations, denoted ${\hat{L}}_{W}\left(\lambda \right)$, was derived by filtering out sun glint in the $L_{T}\left(\lambda \right)$ data. The C-AERO instrument suite enabled rapid $L_{T}$ sampling, at 15 or 30 Hz depending on the acquisition date, for superior glint discretization (and subsequent rejection). The sky reflection was then removed based on a spectral reflectance model depending on the viewing geometry (i.e., pointing angle of the radiometers) and true wind speed (*W*) as measured on the aircraft (removing geometrical terms for brevity):

(4) $${\hat{L}}_{W}\left(\lambda \right)\;=\;L_{T}\left(\lambda \right)\;-\;\rho \left(\lambda ,W\right)L_{i}\left(\lambda \right),$$

where ρ(λ,W) is the water surface reflectance and is obtained from look-up tables based on the surface roughness as parameterized by *W* [29]. The derivation of ${\hat{L}}_{W}$ is based on 15 s flight segments wherein the sun glint filter selects the lowest 5% of the data in terms of radiance in the NIR bands, which are used as a temporal mask for all other wavebands [30]. This very high rejection rate (95% of the data recorded during each flight segment) was adopted for the processing of all airborne data.

In general, ${\tilde{L}}_{W}$ (1) is not exactly equivalent to ${\hat{L}}_{W}$ (4), because $L_{T}$ is not obtained at the same nadir viewing angle as $L_{u}$ (the former is at 40${}^{\circ }$ and the latter is at 0${}^{\circ }$). Consequently, computing normalized forms (2)–(3) from (4) are similarly incorrect. A bidirectional correction for the viewing geometry is not applied, because the dataset used herein includes Case 2—waters with a turbidity range for which the needed bidirectional terms [31] are a challenge exceeding the scope of the present study, e.g., [32]. In addition, algorithm development (Section 2.7) is based on in-water data not requiring correction, and above-water data are only used to demonstrate applicability for an airborne (remote sensing) platform.

Two-dimensional survey maps were generated from the C-AERO observations using a spatial interpolation scheme based on the natural neighbor technique provided by Sibson [33]. For approximately homogenous targets, e.g., a clear-water lake, the spatial variability in remote $a_{\mathrm{CDOM}}\left(440\right)$ estimates using C-AERO observations was quantified using the coefficient of variation (CV). The CV was calculated as the ratio of the standard deviation to the mean and expressed herein as a percentage.

### 2.6. Water Sample Analyses

The determination of in-water constituents typically involves preservation of a water sample (as noted above) and subsequent laboratory analysis as described by The Protocols. Complete details of water sample analyses are provided in Hooker et al. [13], so only a brief summary is provided here. Three optical profiles were obtained in rapid repetition, which were followed by collecting a volume of surface water. Duplicate or triplicate water samples were collected for all coastal and inland waters. For open-ocean campaigns in the Arctic (about 5% of the data herein), a single seawater sample was collected, with some duplicates for quality assurance. Following community protocols, selected volumes of each water sample were filtered. The filter and filtrate were stored and then analyzed in the laboratory, except for some Arctic samples (about 3% of the dataset), wherein the analysis was done onboard ship.

The filter was analyzed to determine the concentration of phytoplankton pigments with high performance liquid chromatography (HPLC) using the [34] C${}_{8}$ column method or slight modifications thereof. The total chlorophyll *a* concentration, [TChl *a*], was computed as the sum of monovinyl and divinyl chlorophyll *a*, plus applicable allomers, epimers, and degradation products (e.g., chlorophyllide *a*). The dynamic range of [TChl *a*] spanned more than three decades—i.e., the oligotrophic, mesotrophic, and eutrophic regimes (0.056–67.484 mg m${}^{-3}$)—and further established the global perspective adopted herein.

The filtrate was used to determine the absorption spectrum of CDOM using a spectrophotometer [13,35] or UltraPath liquid waveguide [13,36]. The blank-corrected absorbance spectrum was baseline-corrected and then converted to the absorption coefficient by including the path length. A single absorbance analysis was generally carried out for the samples collected in the open ocean, whereas duplicate and occasionally triplicate analyses were conducted for the coastal and inland water samples. The different methods used in this study for determining CDOM absorption do not influence the results as shown by the [13] sensitivity analysis.

### 2.7. Algorithmic Approach and Cross-Validation

The algorithmic approach adopted here follows from Hooker et al. [12,13], wherein spectral information from outside the VIS domain is used, while maintaining an ability to exploit legacy data by also including a VIS option. Both one- and two-channel $K_{d}\left(\lambda \right)$ and ${\left[L_{W}\left(\lambda \right)\right]}_{N}$ empirical algorithms are used to estimate $a_{\mathrm{CDOM}}\left(440\right)$, which are based on a global dynamic range of optical data and contemporaneous water sample analyses. To mitigate the confounding influences of phytoplankton, wavelength choices are restricted to the shortest and longest available, i.e., the UV and NIR next-generation end members plus the blue and red legacy end members. The $K_{d}\left(\lambda \right)$ algorithms are anticipated for in-water applications and the ${\left[L_{W}\left(\lambda \right)\right]}_{N}$ algorithms for above-water (remote sensing) applications.

The efficacy of the one- and two-band algorithms was evaluated using 10,000 cross-validation replications, in which the dataset was randomly partitioned into validation and fitting subsets, using a 20% and 80% split, respectively, and based on unique sampling stations. The total number of observations within the validation and fitting subsets within each iteration are defined as $N_{V}$ and $N_{F}$, respectively, and may vary between iterations based on differences in the number of observations collected at each sampling station (recalling that in general three optical profiles were obtained at each water sampling station).

Median statistics from the 10,000 cross-validation iterations were obtained using the coefficient of determination ($R^{2}$) based on log-transformed values plus three other measures. The other statistics are the root mean square difference (RMSD), the mean absolute difference (MAD) or mean absolute error (MAE), depending on the literature, plus the mean bias (MBIAS). The RMSD follows a standard statistical formulation [9], and both the MAD and MBIAS were derived following Seegers et al. [37], as follows:

(5) $$\mathrm{RMSD}\;=\;{\left[\frac{1}{N_{V}}\sum_{i=1}^{N_{V}}{\left(X_{i}-Y_{i}\right)}^{2}\right]}^{1/2},$$

(6) $$\mathrm{MAD}\;=\;10\;\hat{\;}\left[\frac{1}{N_{V}}\sum_{i=1}^{N_{V}}\left|\log_{10}\left(X_{i}\right)\;-\;\log_{10}\left(Y_{i}\right)\right|\right],$$

and

(7) $$\mathrm{MBIAS}\;=\;10\;\hat{\;}\left[\frac{1}{N_{V}}\sum_{i=1}^{N_{V}}\left(\log_{10}\left(X_{i}\right)\;-\;\log_{10}\left(Y_{i}\right)\right)\right],$$

where *i* is an index variable ($i=1,\;2,\;3\ldots N_{V}$), and *X* is the fitted CDOM variable and *Y* is the in situ CDOM variable within the $N_{V}$ subset. The RMSD allows comparison of the results presented herein with prior applicable studies, e.g., [12,13].

The MAD variable is a measure of accuracy and is always greater than or equal to unity, wherein a value of 1.500 indicates a relative measurement error of 50.0%, i.e., the measurement error in percent is obtained by subtracting 1 and multiplying by 100. The MBIAS can be greater than, less than, or equal to 1. An MBIAS value of 1.200 indicates that the fit is on average 20.0% greater than the in situ observations. The optimal result for both variables is a unity value, which indicates zero measurement error (MAD) and zero bias (MBIAS), as appropriate. Unity values are rare for algorithms based on in situ variables with environmentally induced variance, so whether or not an algorithm is fit for its intended purpose requires thresholds for the MAD and MBIAS variables, wherein if they exceed the respective thresholds, the algorithm is not deemed fit for its intended purpose. Appropriate thresholds are selected for this study by comparing the algorithm cross-validation statistics with those reported for algorithms routinely used in the community of practice. For example, Seegers et al. [37] reported MAD values for satellite observations spanning a large dynamic range in [TChl *a*], as a function of trophic levels, as follows: oligotrophic 47–82%, mesotrophic 52–58%, and eutrophic 62–105%, or an overall value of 61–76%. Considering that Seegers et al. [37] did not include extreme values, shallow coastal, or inland waters, the 76% overall MAD value may be considered a defensible—but possibly underestimated—threshold for establishing whether a method provides a useful environmental observation, hereafter termed *fit for purpose*.

### 2.8. Algorithmic Testing with Independent Archival Data

The NASA bio-Optical Marine Algorithm Dataset (NOMAD) v2.a [38] is a quality assured subset of a larger data archive established at the start of the Sea-viewing Wide Field-of-view Sensor (SeaWiFS) satellite mission [39]. The larger repository is called the SeaWiFS Bio-optical Archive and Storage System (SeaBASS) and is described by Hooker et al. [40]. Contemporaneous $a_{\mathrm{CDOM}}\left(440\right)$ plus UV and NIR radiometric data products, as used for a significant part of the spectral domains considered in this study, are not included within the NOMAD database.

The NOMAD database, however, includes radiometric data products spanning legacy VIS wavelengths plus the contemporaneous dissolved (Gelbstoff) spectral absorption coefficient at 443 nm, $a_{g}\left(443\right)$, spanning approximately 0.001–1.116 m${}^{-1}$. Following Röttgers and Doerffer [41], the latter is functionally equivalent to $a_{\mathrm{CDOM}}\left(443\right)$. The consequences of the 3 nm shift in wavelength for $a_{g}\left(443\right)$ with respect to $a_{\mathrm{CDOM}}\left(440\right)$ are considered negligible for a generalized inquiry involving legacy optical data for the following reasons: (a) the fixed wavelengths involved have 10 nm bandwidths, and (b) there are multiple sources of uncertainties in the derived radiometric data products of equal or greater importance [12], e.g., pressure tares, aperture depth offsets, dark currents, wave-focusing effects, etc.

Unlike in the C-OPS cross-validation analysis, in which the $N_{F}$ and $N_{V}$ dataset partitions were based on stations rather than individual observations, each NOMAD data point was treated as an independent observation, due to the lesser degree of ancillary information recorded in the NOMAD repository compared to the C-OPS dataset. Potential interdependencies between the fitting and validation NOMAD dataset partitions were considered negligible for the purpose of this work, because NOMAD is included to provide an independent algorithmic perspective, and the C-OPS dataset provides the greatest number of relevant wavelengths and the highest quality data for algorithm fitting and validation.

## 3. Results

The focus of this study is to establish the efficacy of one-band algorithms to derive $a_{\mathrm{CDOM}}\left(440\right)$ from ${\left[L_{W}\left(\lambda \right)\right]}_{N}$ or $K_{d}\left(\lambda \right)$ data products that can be used with notional concepts for appropriate new sensors presented in Section 2 and expanded upon in Section 4.2. In addition to presenting the one-band algorithms, the following sections document the efficacy using the following: (a) a cross-validation analysis (Section 2.7), (b) performance comparisons to established two-band algorithms, and (c) validation based on independent archival data (Section 2.8), as applicable (i.e., to the spectral extent possible).

### 3.1. C-OPS One-Band $K_{d}\left(\lambda \right)$ Algorithms

**Figure 3:**
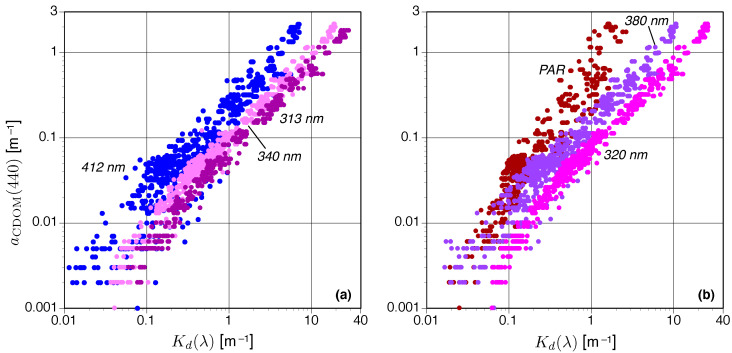
The in situ observations for the one-band C-OPS $K_{d}\left(\lambda \right)$ algorithms to derive $a_{\mathrm{CDOM}}\left(440\right)$ with the spectral bands chosen to emphasize both a next-generation (UV) and legacy (blue domain) perspective: (**a**) the data for 313, 340, and 412 nm (left panel), and (**b**) the data for 320 and 380 nm plus PAR (right panel).

The derived $K_{d}\left(\lambda \right)$ values used in this study are shown in Figure 3, wherein two panels are used to display all applicable spectral data products. The figure shows $K_{d}$ values for a particular $a_{\mathrm{CDOM}}\left(440\right)$ value generally decrease with increasing wavelength, with $K_{d}\left(PAR\right)$ most similar to $K_{d}\left(412\right)$. Variance (dispersion) in the data increases with increasing wavelength—notably in clearer waters—which is likely due, in part, to wave-focusing effects. The latter means some longer-wavelength observations at low $a_{\mathrm{CDOM}}\left(440\right)$ values are obscured by the shorter-wavelength data in Figure 3, but the statistical descriptions of data distribution and fitting presented below are not affected.

Figure 3 also shows the shortest wavelengths (313, 320, and 340 nm) exhibit a significant linear response as a function of $a_{\mathrm{CDOM}}\left(440\right)$ as revealed by the log-scale curvature towards an apparent constant $K_{d}\left(\lambda \right)$ value—indicative of the anticipated pure-water value, $K_{w}\left(\lambda \right)$—at low $a_{\mathrm{CDOM}}\left(440\right)$ values. The longer wavelengths (380 and 412 nm plus PAR), however, do not exhibit a distinctive tail at low concentrations. Instead, the log-transformed data exhibit a rather constant slope, which evidences adherence to a power law. These observations establish likely functional forms for the fits, which are confirmed by the statistical parameters derived by performing both types of fitting and selecting the best performance. The best performance is associated with maximal $R^{2}$, minimal MAD, and near-unity MBIAS values (Section 2.4).

The efficacies of the final one-band $K_{d}\left(\lambda \right)$ algorithms are presented in Table 1, wherein only the best performing algorithm type is displayed and the type is identified by the resulting fitted equation (linear or power). The $R^{2}$ values exhibit significant correlation for all wavelengths and span values of 0.877–0.986. The shortest wavelengths (313 and 320 nm) have approximately 99% of the variance explained by linear fits, which is in keeping with Hooker et al. (2020). The MAD is best interpreted by recalling a value of 1.50 indicates a relative measurement error of 50%. Consequently, the measurement errors provided in Table 1 are less than 50% for all discrete wavelengths and less than 25% for the three shortest wavelengths; the PAR results have a measurement error slightly larger than 50%. All the fits in Table 1 have a small absolute bias of approximately 0.1–0.3% (a negative value indicates the algorithm under-predicts the in situ variable). The rapid cycle times of optical profiles obtained with the C-PrOPS accessory usually result in an environmental variability less than 5% even in heterogenous water bodies, so the MBIAS results indicate the fits are unbiased to within environmental uncertainty.

### 3.2. C-OPS and NOMAD One-Band ${\left[L_{W}\left(\lambda \right)\right]}_{N}$ Algorithms

**Figure 4:**
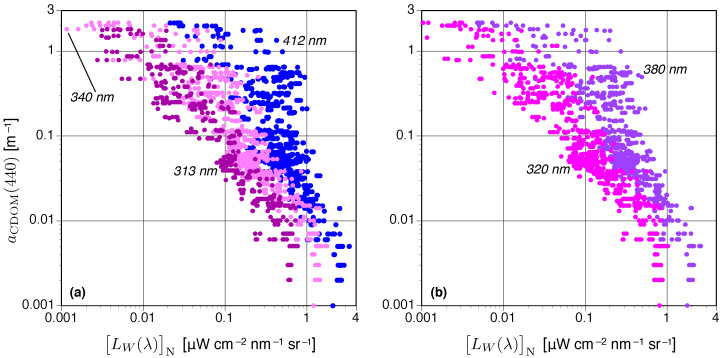
The in situ observations for the one-band C-OPS ${\left[L_{W}\left(\lambda \right)\right]}_{N}$ algorithms to derive $a_{\mathrm{CDOM}}\left(440\right)$ emphasizing a next-generation (UV) and legacy (blue domain) perspective: (**a**) the data for 313, 340, and 412 nm (left panel), and (**b**) the data for 320, and 380 nm (right panel).

The C-OPS ${\left[L_{W}\left(\lambda \right)\right]}_{N}$ data products used in this study are shown in Figure 4 and follow the format established in Figure 3. Again, the presentation format means some longer-wavelength observations are obscured by the shorter-wavelength data, but the statistical descriptions of data distribution and fitting presented below are not affected. A comparison of Figure 3 and Figure 4 reveals the following:

1. The corresponding ${\left[L_{W}\left(\lambda \right)\right]}_{N}$ values for a particular $a_{\mathrm{CDOM}}\left(440\right)$ value decrease with decreasing wavelength, whereas $K_{d}\left(\lambda \right)$ values increase with decreasing wavelength;
2. The dispersion (or variance) in ${\left[L_{W}\left(\lambda \right)\right]}_{N}$ values as a function of $a_{\mathrm{CDOM}}\left(440\right)$ increases with increasing turbidity, whereas the corresponding dispersion in $K_{d}\left(\lambda \right)$ increases with increasing clarity (so they are opposite);
3. The ${\left[L_{W}\left(\lambda \right)\right]}_{N}$ data generally exhibit more dispersion than the corresponding $K_{d}\left(\lambda \right)$ data; and
4. Both datasets exhibit similar sensitivity as measured by the range of values corresponding to the *x*-axis, although the ${\left[L_{W}\left(\lambda \right)\right]}_{N}$ data artificially appear to have a slightly larger range due to the increased variance for high $a_{\mathrm{CDOM}}\left(440\right)$ values.

The opposing trends in dispersion are likely due to $K_{d}\left(\lambda \right)$ being more sensitive to wave focusing in clear waters and ${\left[L_{W}\left(\lambda \right)\right]}_{N}$ being more sensitive to broad-spectrum brightness effects (e.g., due to an elevated nonalgal particle concentration) in turbid waters. As optical complexity increases, upwelled radiant flux at the shortest wavelengths is often significantly modified, so small absolute differences in the magnitude of derived data products lead to large dispersions or variance [20]. Both Houskeeper et al. [9] and Hooker et al. [13] found that the correlation between $a_{\mathrm{CDOM}}\left(440\right)$ and above- or in-water data products, respectively, was the most significant at the spectral end members. Both studies also showed the algorithmic relationships based on spectral end members were the most robust cross a global range in water bodies, e.g., waters varying in algal pigmentation, spectral slope of $a_{\mathrm{CDOM}}\left(440\right)$, or suspended particles.

The median efficacies of the final one-band ${\left[L_{W}\left(\lambda \right)\right]}_{N}$ algorithms plus the NOMAD results are presented in Table 2, wherein only the best performing algorithm type (linear or power) is displayed for each data product. In all cases, the best results are always obtained with power fits. The $R^{2}$ values exhibit the most significant correlation (i.e., more than 80% of the variance explained) for the shortest wavelengths (313, 320, and 340 nm), with $R^{2}$ spanning 0.825–0.876. All $R^{2}$ and MAD results in Table 2 are less than and greater than, respectively, the corresponding $K_{d}\left(\lambda \right)$ fits in Table 1. The measurement uncertainties in Table 2 are more than 50% for all wavelengths and exceed 76% (MAD) for the two longest wavelengths (380 and 412 nm), including the NOMAD results. For both the $K_{d}\left(\lambda \right)$ and ${\left[L_{W}\left(\lambda \right)\right]}_{N}$ fits, the $R^{2}$ values decrease with increasing wavelength, and the MAD values increase with increasing wavelength (with a small exception for 313 nm, presumably because of the lesser amount of data at this wavelength). All the curve fits in Table 2 have a negligible bias of approximately 0.1–0.7%.

Although the performance of the 412 nm data in Table 2 is the worst, the separate realizations using C-OPS and NOMAD data provide a similar algorithmic perspective. The exponential slope coefficient for the NOMAD fit is significantly less negative compared to the C-OPS fit, consistent with the two-waveband ${\left[L_{W}\left(\lambda \right)\right]}_{N}$ algorithms presented in Houskeeper et al. [9], which noted that differences in the range of $a_{\mathrm{CDOM}}\left(440\right)$ between the NOMAD and C-OPS datasets would result in differences in slopes due to fitting or due to potential log-space nonlinearities in the algorithmic relationship. The NOMAD $R^{2}$ value is also poorer, which indicates the NOMAD data have more dispersion than the C-OPS equivalent. Given that NOMAD was created by contributions from a multitude of practitioners, whereas the C-OPS data were created by one practitioner, increased variance in the former is anticipated. Algorithms for additional ${\left[L_{W}\left(\lambda \right)\right]}_{N}$ wavelengths cannot be similarly validated because NOMAD does not include the shorter wavelengths of interest.

### 3.3. C-OPS Two-Band (Band-Ratio) $K_{d}\left(\lambda \right)$ and ${\left[L_{W}\left(\lambda \right)\right]}_{N}$ Algorithms

Two-band algorithms are considered herein for two reasons: (a) band-ratio formulations can partially cancel out broadband sources of variance, e.g., wave focusing, that can increase the variance in data products and, thus, degrade algorithm performance; and (b) for legacy datasets, wherein wavelength choices are usually limited to the VIS domain, the use of a band ratio might provide improved algorithmic performance compared to a single channel. The latter possibly safeguards significant research opportunities, because it might not be possible to detect long-term changes or trends in aquatic ecosystems without legacy datasets.

The original [12] linear equation for the $K_{d}\left(320\right)/K_{d}\left(780\right)$ algorithm was y=0.2556x−0.0030. The Hooker et al. [13] study confirmed the global application of this algorithm, and for a maximally expanded dataset composed of conservative and near-conservative water bodies including analysis of independent (Chesapeake Bay) data, the agreement was to within 1%. The corresponding fit in Table 3 has the same intercept as Hooker et al. [12] and the slope agrees to within approximately 1%, so the two are considered functionally equivalent for the purposes of this study.

The efficacy of the final band-ratio algorithms are presented in Table 3, wherein only the best performing algorithm type (linear or power) is presented. The $K_{d}\left(320\right)/K_{d}\left(780\right)$ results are distinguished by near-unity $R^{2}$ and MBIAS values and a very low MAD value of 7.5%. The $R^{2}$ and MAD values are superior to any of the single-channel $K_{d}\left(\lambda \right)$ results in Table 1, and the MBIAS results are generally better. The $K_{d}\left(412\right)/K_{d}\left(670\right)$ results in Table 3 are rather similar to the $K_{d}\left(412\right)$ results in Table 1, although the band-ratio MAD and $R^{2}$ values are both somewhat lower and higher, respectively, and the MBIAS value is slightly improved.

The ${\left[L_{W}\left(\lambda \right)\right]}_{N}$ algorithm results shown in Table 3 have high $R^{2}$ values of 0.913 and 0.924, the MAD values are similarly equal (close to 40%), and the MBIAS value is close to zero or nonetheless rather small (−0.7%). The $R^{2}$ and MAD values are superior to any of the single-channel ${\left[L_{W}\left(\lambda \right)\right]}_{N}$ results in Table 2, and the MBIAS results are generally better (albeit MBIAS is near unity for all algorithms presented herein). When comparing the algorithm validation results within Table 1, Table 2 and Table 3, the distinguishing metrics are associated with goodness-of-fit or accuracy, i.e., $R^{2}$, RMSD, and MAD.

In regard to comparing Table 1, Table 2 and Table 3 and the assessments of accuracy therein, the following generalizations emerge:

1. The most accurate algorithms include the shortest (UV) wavelength(s);
2. The $K_{d}\left(\lambda \right)$ algorithms are more accurate than the ${\left[L_{W}\left(\lambda \right)\right]}_{N}$ equivalents (although the former PAR algorithm has no equivalent in the latter);
3. The $K_{d}\left(\mathrm{PAR}\right)$ algorithm is approximately as accurate or more accurate than the ${\left[L_{W}\left(\lambda \right)\right]}_{N}$ algorithms;
4. The spectrally shortest one-band $K_{d}\left(\lambda \right)$ algorithms (i.e., 313, 320, and 340 nm) are more accurate than the best (band-ratio) ${\left[L_{W}\left(\lambda \right)\right]}_{N}$ algorithms; and
5. The most accurate algorithm is the $K_{d}\left(320\right)/K_{d}\left(780\right)$ band ratio [12,13].

The fifth assessment requires a qualifier, because the amount of data collected at 313 nm was less than collected at 320 nm and the difference in performance for the former with respect to the latter was not substantially worse (Table 1).

From the vantage of above- versus in-water perspectives, the in-water measurements used to derive the algorithms were challenged at 780 nm relative to 670 nm, because of the following: (a) lower signal levels and higher attenuation for the NIR as opposed to the red domain, and (b) a typically deeper start of the $L_{u}\left(z,\lambda \right)$ data versus $E_{d}\left(z,\lambda \right)$ data within the extrapolation interval (set by $z_{1}$ and $z_{2}$) used to derive in-water data products. An in-depth comparison of applying above-water algorithms was documented in Houskeeper et al. [9], which found that the most spectrally separate above-water algorithms produced the most robust statistics across a global range in water bodies.

### 3.4. C-AERO One-Band Results Applied to Lake Tahoe Observations

**Figure 5:**
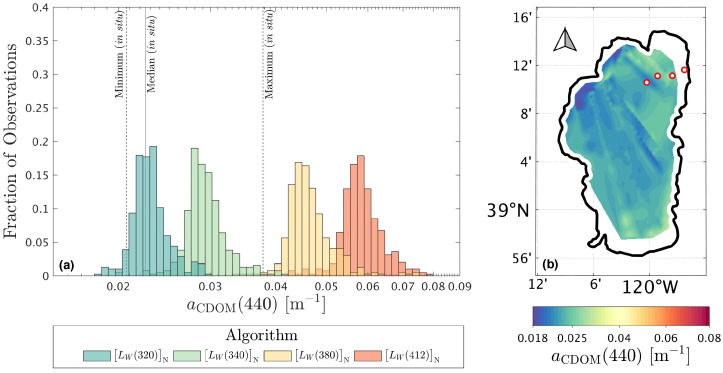
The one-band ${\left[L_{W}\left(\lambda \right)\right]}_{N}$ algorithms to derive $a_{\mathrm{CDOM}}\left(440\right)$ at LT: (**a**) The distribution of remotely estimated $a_{\mathrm{CDOM}}\left(440\right)$ results using the color-coded algorithms (none of which are significantly biased across the global dataset) with respect to the in situ $a_{\mathrm{CDOM}}\left(440\right)$ values indicated by labeled dashed and solid lines (left); and (**b**) the spatial interpolation of the ${\left[L_{W}\left(320\right)\right]}_{N}$ algorithm applied to the airborne observations, with the in situ sampling sites indicated as red circles (right).

Airborne ${\left[L_{W}\left(\lambda \right)\right]}_{N}$ observations of Lake Tahoe (LT), located on the California-Nevada border, were obtained using C-AERO radiometers mounted on a Twin Otter fixed-wing (propeller) aircraft flying at LSA. Coincident water samples during aircraft overflights were obtained on the eastern edge of the lake from a small boat. The left panel of Figure 5 demonstrates that the $a_{\mathrm{CDOM}}\left(440\right)$ estimated from the ${\left[L_{W}\left(\lambda \right)\right]}_{N}$ algorithms using the spectrally shortest available wavelengths (i.e., 320 and 340 nm) predominantly overlap the range of in situ sampling, while the longest wavelength ${\left[L_{W}\left(\lambda \right)\right]}_{N}$ algorithms (i.e., 380 and 412 nm) do not and, thus, over predict $a_{\mathrm{CDOM}}\left(440\right)$. Recalling that the derived algorithms show no significant bias across the global range of water bodies, the local over prediction for the longer-wavelength algorithms when applied to a single, clear, substantially homogeneous sampling area (i.e., LT) may result from linear regression, as well as increasing log-space nonlinearities in the relationship between ${\left[L_{W}\left(\lambda \right)\right]}_{N}$ and $a_{\mathrm{CDOM}}\left(440\right)$ as wavelength increases, which is evident for the ${\left[L_{W}\left(380\right)\right]}_{N}$ and ${\left[L_{W}\left(412\right)\right]}_{N}$ relationships shown in Figure 4.

For the better performing shorter wavelength algorithms, the potential to survey LT using a drone with a one-band radiometer is demonstrated in the right panel of Figure 5, which shows $a_{\mathrm{CDOM}}\left(440\right)$ estimated using the ${\left[L_{W}\left(320\right)\right]}_{N}$ algorithm, and spatially interpolated using the natural neighbor method [33]. The color scheme was chosen to emphasize uncertainties in the remote estimation of $a_{\mathrm{CDOM}}\left(440\right)$ using a one-band, above-water algorithm, but the spatial variability is low (CV =8.8%) relative to the algorithm MAD uncertainty estimated in Table 2 (MAD =58.8%). Low inter-pixel variability combined with good agreement to in situ water sampling is in part due to the bio-optical characteristics of LT, which is oligotrophic and optically simple, and therefore more conducive to a one-band, above-water algorithmic perspective.

## 4. Discussion

Using the maximum, combined water type MAD value of 76%—proposed in Section 2.7 based on the performance of algorithms commonly used by the community of practice [37]—as an overall threshold to determine whether an algorithm is fit for purpose, all the $K_{d}$ and both types of band-ratio algorithms are compliant. The only algorithms that fail the MAD 76% threshold are the ${\left[L_{W}\left(380\right)\right]}_{N}$ and ${\left[L_{W}\left(412\right)\right]}_{N}$ one-band algorithms (Table 2). From the point of view of adopting a one-band approach for deriving $a_{\mathrm{CDOM}}\left(440\right)$, this restriction is minor, because the compliant bands are equally easy to implement as the noncompliant bands. A possible application wherein the restriction is inconvenient and unavoidable is with archival data, wherein there are perhaps no other wavelength alternatives, e.g., legacy measurements often have 412 nm as the shortest wavelength.

Another consideration in algorithm selection is the advantage of minimizing bias. For the most accurate results in Seegers et al. [37], which considered matchups using algorithmic results from satellite data and in situ water samples, the MBIAS results spanned −20% to 39%. For the algorithmic results presented here, which are based on in-water optical observations and contemporaneous water samples, MBIAS values for all algorithms were to within the environmental uncertainty and ranged from −0.3% to 0.9%. Applicable reasons for the latter negligible bias are as follows: (a) the in-water sampling strictly adhered to The Protocols [19]; (b) the optical observations were obtained with consistent and advanced instrumentation, e.g., C-PrOPS was used in almost 97% of the data [13]; and (c) all data were acquired and processed with the same advanced software [15]. These three points are generically applicable to remote sensing data—i.e., The Protocols are followed plus advanced instrumentation and software are used—but satellite data also require atmospheric correction, which in situ optical data do not. This point is counterbalanced, to some degree, by the use of quality control measures when determining matchups (e.g., filtering, binning, and median statistics), whereas the study presented here was based on accepting data regardless of variance measures—including within optically complex waters.

### 4.1. One- versus Two-Band Algorithms and Wavelength Selection

In regard to the algorithms considered herein, the two-channel $K_{d}\left(\lambda \right)$ algorithm based on Hooker et al. [13], which uses 320 and 780 nm, provided the most accurate estimation for $a_{\mathrm{CDOM}}\left(440\right)$, with a MAD value of 7.5% and approximately uniform log-scale residuals across the full range in $a_{\mathrm{CDOM}}\left(440\right)$. The single-channel $K_{d}\left(\lambda \right)$ algorithms for the shorter wavelengths (i.e., 313, 320, and 340 nm) provided slightly less accurate estimation of $a_{\mathrm{CDOM}}\left(440\right)$, with a MAD value of 17.0%, 15.4%, and 20.1%, and also produced approximately uniform log-scale residuals across the full range in $a_{\mathrm{CDOM}}\left(440\right)$. The small decrease in accuracy for one- versus two-band $K_{d}\left(\lambda \right)$ algorithms indicates one-band instruments are potentially useful for research scenarios in which lowering the cost, power budget, or data transmission and storage requirements warrants a small reduction in algorithmic accuracy. The consistent performance across oligotrophic, mesotrophic, and eutrophic regimes supports the use of one- and two-channel $K_{d}\left(\lambda \right)$ algorithms—in which the principal wavelength is within the UV domain—for obtaining consistent observations at global scales.

In-water $K_{d}\left(\lambda \right)$ algorithms based on longer wavelengths (i.e., 380 and 412 nm) are more degraded relative to the two-band algorithms, with the greatest MAD value of 53.6% produced by the $K_{d}\left(PAR\right)$ algorithm, which arguably has the longest wavelength responsivity, because it spans the VIS domain. PAR instruments, however, have been—and continue to be—routinely deployed in many existing oceanographic observing systems. Consequently, the potential applications for PAR data products and an $a_{\mathrm{CDOM}}\left(440\right)$ algorithm based on $K_{d}\left(\mathrm{PAR}\right)$ are useful.

For the ${\left[L_{W}\left(\lambda \right)\right]}_{N}$ algorithms, the addition of a second, NIR wavelength conferred on average a greater improvement to algorithm accuracy than to the $K_{d}\left(\lambda \right)$ algorithms and improved the robustness of the ${\left[L_{W}\left(\lambda \right)\right]}_{N}$ approach across global waters, or across oligotrophic, mesotrophic, and eutrophic ecosystems. For example, the two-channel ${\left[L_{W}\left(\lambda \right)\right]}_{N}$ algorithm based on Houskeeper et al. [9] using 320 and 780 nm produced a MAD value of 41.4%, compared to 58.8% for the best performing one-channel ${\left[L_{W}\left(\lambda \right)\right]}_{N}$ algorithm evaluated herein, which was based on ${\left[L_{W}\left(320\right)\right]}_{N}$. The two-channel ${\left[L_{W}\left(\lambda \right)\right]}_{N}$ algorithm was applicable to global waters, i.e., algorithm log-scale residuals were approximately uniform across the global range in $a_{\mathrm{CDOM}}\left(440\right)$, but the single-channel ${\left[L_{W}\left(\lambda \right)\right]}_{N}$ algorithms were significantly degraded in higher $a_{\mathrm{CDOM}}\left(440\right)$ waters, which is consistent with the findings of Hooker et al. [20], in which ${\left[L_{W}\left(\lambda \right)\right]}_{N}$ was most sensitive to increasing optical complexity within the UV and NIR domains.

Using $a_{\mathrm{CDOM}}\left(440\right)$ of 0.1$\;m^{-1}$ as an approximate partition separating global waters into oceanic waters and coastal or inland waters, the findings presented herein indicate that one-band approaches using either above- or in-water observations are appropriate for observations of oceanic waters, with greater algorithmic accuracy for in-water compared to above-water approaches. In the higher $a_{\mathrm{CDOM}}\left(440\right)$ partition corresponding to coastal and inland waters, above-water algorithms using one band were less useful based on increased variance in the algorithm residuals. For operational considerations, however, above-water observations are in some circumstances more favorable for coastal and inland water observation because coastal and inland waters are generally more spatially heterogeneous (drifting floats are unlikely to sample across frontal boundaries) and may contain significant and uncharted navigational hazards in comparison to oceanic waters.

The Houskeeper et al. [9] study found that two-channel ${\left[L_{W}\left(\lambda \right)\right]}_{N}$ algorithms that use an expansive spectral range (i.e., UV to NIR) improve estimation of $a_{\mathrm{CDOM}}\left(440\right)$ across a global range in water bodies, including coastal and inland waters. The more expansive spectral range was also found to produce algorithms that were more robust to natural variability in the aquatic constituents. The expanded dataset considered herein yielded results consistent with the earlier, more limited dataset [9] and also supports the use of a two-channel ${\left[L_{W}\left(\lambda \right)\right]}_{N}$ approach—in which the principal wavelength is within the UV domain—for obtaining globally consistent observations. This two-channel, above-water approach does not necessarily require significant increases in the weight or power requirements for above-water systems, and data storage or transmission constraints can be managed with onboard preprocessing. For example, information collected from a two-band sensor could be reduced to a single channel by computing a band ratio before storage or transmission of the data, although there would be a commensurate loss in reconstructing the performance of each channel (if desirable).

### 4.2. Technology Constraints and Opportunities

The constraints and opportunities technology places on determining whether to use $K_{d}\left(\lambda \right)$ or ${\left[L_{W}\left(\lambda \right)\right]}_{N}$ algorithms naturally sorts the discussion into the following: (a) $K_{d}\left(\lambda \right)$ is best for in-water platforms (no self-shading correction but cannot be measured directly from above the water surface), and (b) ${\left[L_{W}\left(\lambda \right)\right]}_{N}$ is the most advantageous for above-water platforms (self-shading effects are easily avoided, but supporting measurements are needed). Consequently, hereafter, the $K_{d}\left(\lambda \right)$ perspective is applied only to AUVs, and the ${\left[L_{W}\left(\lambda \right)\right]}_{N}$ perspective is restricted to UAVs (albeit, nonetheless possible for AUVs). In terms of the simplest architecture possible, while satisfying algorithm efficacy (Table 1, Table 2 and Table 3), an AUV or float is anticipated for a one-channel irradiance sensor and a UAV for a two-channel radiance sensor.

The description of deployments for AUVs and UAVs may use similar language that is, in fact, rather different, so the following clarification is made: a long AUV deployment lasts weeks and months, whereas a long UAV deployment lasts hours. Although UAV designs capable of deploying for periods as long as AUVs are feasible, the community of practice supporting the algorithmic research presented here is poised to exploit profiling floats for in-water measurements and drones for above-water observations. In both UAV and AUV deployments, the one- or two-channel algorithms enabled by the sensors presented herein provide the advantages of reductions in power consumption, mass, data storage, and cost relative to most multispectral or hyperspectral radiometers, with an application-dependent disadvantage of less spectral information being acquired from the targets. Additionally, the simplicity of the sensors presented allow opportunistic integration into existing UAV and AUV architectures in scenarios where budgetary or time constraints preclude extensive customization. The actual range of conditions (e.g., depth and duration) for which useful data may be acquired is limited by the temporal and spatial nature of a specific deployment and the integration platform, but the low-power requirements in conjunction with the extended dynamic range relative to legacy sensors is anticipated to enhance the applicability of the sensors described herein both in mission duration and observable flux levels relative to legacy technologies.

The difference in deployment time scales for AUVs and UAVs means bio-fouling is an anticipated problem for the former but not the latter (although possible due to random episodic events, which cannot be prevented). Polytetrafluoroethylene (PTFE) is an ideal material to construct diffusers for measuring solar irradiance over the UV–VIS range, because it is hydrophobic and can be formed into an optimal shape with a compliant cosine response (per The Protocols) for underwater collectors while presenting a well-defined reference plane for calibrations and withstanding the hydrostatic pressure associated with euphotic sampling depths [42]. The same shape with compliant cosine response is suitable for above-water irradiance radiometers. To minimize energy and weight budgets, passive in-water antifouling is preferred, e.g., stowing the AUV at depth well below the 0.1% light level. The hydrostatic pressure at such depths, however, also makes acrylic with sufficient UV transmission an attractive alternative to PTFE.

The above-mentioned technology considerations, plus the results presented in Table 1, Table 2 and Table 3, suggest a single-channel algorithmic approach is viable for an in-water sampling system, but an above-water system requires a band-ratio approach, i.e., two channels, for comparable performance. Although this conclusion provides sampling guidance for a wide diversity of water masses, it does not provide a mechanism for expanding the generation of data products in extremely difficult or unmeasurable environments, e.g., during twilight and moonlight, transitional seasons, and polar winters, plus the deep ocean or very turbid waters. Strategies for increasing the generation of data products for previously difficult or unmeasurable environments include the following: (a) predictive dark current (PDC) correction [28] to account for instrument performance fluctuations (e.g., due to temperature) when signal levels are very low; and (b) pairing a photomultiplier tube (PMT) with a microradiometer to produce a *hybridnamic* sensor, which extends the dynamic range for very dark targets [14].

PDC improves the quality of data products when the signal-to-noise ratio (SNR) is low and degraded by significantly variable instrument performance parameters. This is typically an above-water sampling issue, because airborne platforms can experience large fluctuations in performance variables within a short amount of time (e.g., by changing altitude), whereas an in-water platform usually does not. An exception for in-water platforms is for long-term deployments spanning large distances or during short-term deployments when an instrument system is left on the deck of a ship in sunlight. The latter is an easily avoided problem [19] and is not considered here.

In regard to previous studies and the working hypothesis that one- and two-channel algorithms are suitable for above- and in-water sampling systems, a future research direction of benefit to the community is to pair a PMT with a microradiometer and include PDC characterization for both. This combination extends the total number of decades in the linear dynamic range from 10 to approximately 14 decades [14]. A PMT has an operational vulnerability, however, if it is exposed to very bright light sources, which can potentially degrade sensitivity and stability. The construction of a PMT-microradiometer dyad allows the power to the PMT to be automatically controlled (on or off) based on the simultaneous observations of the microradiometer photodiode, thereby significantly mitigating an overexposure risk to the PMT. Additional safety margin can be implemented by including a temporal trend test to ensure illumination anomalies are discounted.

**Figure 6:**
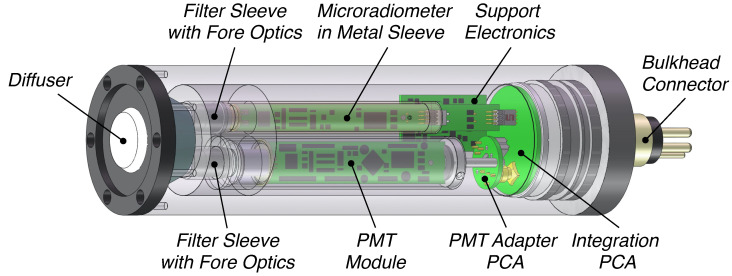
Paired PMT and microradiometer detector technologies in a irradiance sensor dyad (PAR or fixed wavelength) with a 2.0 in (5.1 cm) housing diameter that is 9.5 in (24.1 cm) long, and a total weight of 1.8 lb (0.8 kg). The nominal depth rating is 2000 m, although variations of the design with a greater depth capability are easily fabricated.

The efficacy of the single-channel algorithms when using UV wavelengths further mitigates the PMT overexposure risk because of reduced UV light levels inherent to the solar spectrum (the steepest decline in flux of the solar spectrum is in the UV domain) combined with anticipated and likely problematic research environments (e.g., low solar zenith angles and low transmission through water). An in-water PMT-microradiometer sensor is considered first, because it is the simplest, i.e., based on a single wavelength, for providing a $K_{d}\left(\lambda \right)$ data product in the UV (Table 1). Figure 6 shows a PMT-microradiometer irradiance dyad, wherein the microradiometer components follow directly from Figure 1. The miniature and ruggedized PMT views the underside of the diffuser, but it is slightly longer than the microradiometer. A PMT adapter PCA and integration PCA allow the two detector technologies to function as a single hybridnamic sensor permitting deep sea plus twilight and winter observations, particularly in polar regions.

**Figure 7:**
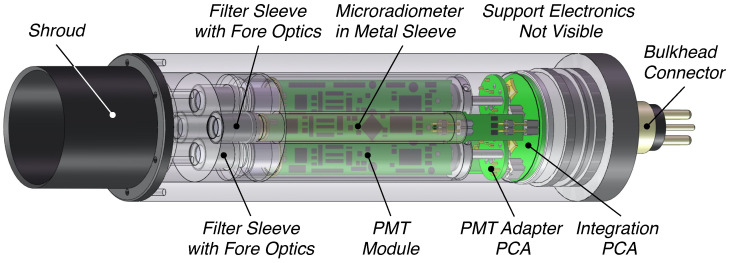
Two paired PMT-microradiometer radiance sensor dyads with a 1.7 in (4.3 cm) diameter and 1.5 in (3.8 cm) long removable shroud attached to a 2.0 in (5.1 cm) diameter housing that is 9.7 in (24.6 cm) long and a total weight of 2.1 lb (1.0 kg). Following from the common architecture in the irradiance dyad, the radiance sensor shown is pressure rated to 2000 m for in-water deployments. Mass reductions exceeding 25% are easily achieved for above-water applications.

A two-channel (fixed wavelength) radiance sensor based on a PMT-microradiometer dyad pair is shown in Figure 7, which has the same housing diameter as the irradiance sensor shown in Figure 6. The two-channel hybridnamic sensor can provide data products—and allow a two-channel algorithm implementation—while observing significantly darker targets than microradiometer technology alone. Observations during twilight and lunar illumination at night are feasible and allow diurnal variations to be studied [14]. In-water use of this hybridnamic sensor has the same benefits and drawbacks as discussed previously, with the PMT benefitting from being downward-pointing to provide protection against excess illumination, regardless of the spectral bandpass.

## 5. Conclusions

Establishing the efficacy of one- and two-band algorithms opens a path to smaller and less expensive sensor requirements (Figure 1 and Figure 2) for the acquisition of data required to derive a useful data product. Based on the methods presented, existing and minimally modified sensors utilizing the same underlying technology to create more advanced hybrids were identified and discussed (Figure 6 and Figure 7). The identified sensors either are, or can readily be, optimized for inclusion on above- and in-water applications (UAV or AUV, respectively), consistent with the paradigm of expanding the volume of observations while reducing the sensor, platform, and personnel costs.

How an individual researcher might exploit the algorithms presented in Table 1, Table 2 and Table 3 depends on the interplay of the factors controlling the research, e.g., environment(s) of interest, legacy needs, above- or in-water sampling, wavelength selection, radiometric configuration, technological constraints, and performance requirements (proposed herein as compliance with the overall MAD fit-for-purpose threshold of 76%). To present the conclusions associated with these six considerations, a one-band algorithm is denoted $\lambda_{1}$ and a two-band algorithm $\lambda_{1}/\lambda_{2}$ (following Table 3).

From an overall perspective, the $K_{d}\left(\lambda \right)$ and ${\left[L_{W}\left(\lambda \right)\right]}_{N}$ algorithms exhibited similar sensitivity as measured by the dynamic range in both data products (Figure 3 and Figure 4). The most accurate algorithms used the shortest (UV) wavelengths with the $K_{d}\left(\lambda \right)$ algorithms being superior and providing the simplest (linear) relationships (Table 1 and Table 3). All the ${\left[L_{W}\left(\lambda \right)\right]}_{N}$ algorithms were nonlinear and performed worse than their $K_{d}\left(\lambda \right)$ equivalents (Table 2 and Table 3). The $K_{d}\left(PAR\right)$ algorithm provided compliant estimates of $a_{\mathrm{CDOM}}\left(440\right)$ (Table 1) and could take advantage of legacy instrumentation or observations.

All the $K_{d}\left(\lambda_{1}\right)$ and $K_{d}\left(\lambda_{1}/\lambda_{2}\right)$ algorithms (Table 1 and Table 3) complied with the proposed MAD fit-for-purpose threshold of 76%, whereas the ${\left[L_{W}\left(\lambda_{1}\right)\right]}_{N}$ algorithms (Table 2) for 380 nm and 412 nm did not (and the NOMAD data did not); the ${\left[L_{W}\left(\lambda_{1}/\lambda_{2}\right)\right]}_{N}$ algorithms, however, were compliant (Table 3). The $\lambda_{1}/\lambda_{2}$ algorithms provided superior performance with respect to their $\lambda_{1}$ principal constituent, e.g., $K_{d}\left(320/780\right)$ and ${\left[L_{W}\left(320/780\right)\right]}_{N}$ were superior to $K_{d}\left(320\right)$ and ${\left[L_{W}\left(320\right)\right]}_{N}$, respectively.

The overall summary results suggest an in-water sampling approach is preferred, because $K_{d}\left(\lambda \right)$ can be obtained. From the perspective of environmental $\lambda_{1}$ sampling objectives, the $K_{d}\left(320\right)$ algorithm provided the best MAD (15.4%) performance, with no significant MBIAS and consistent log-scale performance across open ocean, coastal, and inland waters. Consequently, the $K_{d}\left(320\right)$ algorithm is anticipated to be the best option where in-water $\lambda_{1}$ deployment is suitable (e.g., no navigational hazards or access limitations). A similar result is likely for the $K_{d}\left(313\right)$ algorithm but should be confirmed using more data.

Some of the challenges in sampling coastal and inland waters with an in-water $\lambda_{1}$ system can be overcome with an above-water $\lambda_{1}$ approach, but in these same regions the efficacy of an above-water method also decreases. For example, the variance in ${\left[L_{W}\left(\lambda \right)\right]}_{N}$ increased as a function of $a_{\mathrm{CDOM}}\left(440\right)$ (Figure 4), such that performance was optimal in clearer waters and relatively worse in eutrophic waters. This change in log-scale performance as a function of $a_{\mathrm{CDOM}}\left(440\right)$ was less evident for the shortest wavelengths. The ${\left[L_{W}\left(320\right)\right]}_{N}$ algorithm provided the best $\lambda_{1}$ performance in terms of MAD (58.8%), although the ${\left[L_{W}\left(313\right)\right]}_{N}$ algorithm is also anticipated to produce a robust relationship (but more data is required for confirmation).

Although the same logic is applicable to in-water $\lambda_{1}$ sampling, the more significant variance associated with above-water $\lambda_{1}$ performance can be improved by adopting a $\lambda_{1}/\lambda_{2}$ approach (Table 3). In-water sampling is subject to an additional constraint, because there is an ultimate deployment restriction that is reached when the water depth limits the ability for an in-water system to make profiles. Presently, at a 0.5 m water depth and less, an above-water ${\left[L_{W}\left(\lambda_{1}/\lambda_{2}\right)\right]}_{N}$ algorithm is likely the most practical (assuming turbidity prevents bottom reflection perturbations). Using wavelengths with high attenuation (i.e., the spectral end members) also mitigates bottom reflectance for shallow-water remote sensing. The study of Houskeeper et al. [9] demonstrated robust ${\left[L_{W}\left(\lambda_{1}/\lambda_{2}\right)\right]}_{N}$ log-scale performance across a global range in water bodies, including oligotrophic, mesotrophic, and eutrophic environments, which was confirmed herein with a larger dataset and a fit-for-purpose criterion (Table 3). From an instrument integration perspective, the addition of a second channel does not significantly increase weight or power, because the microradiometer building block is small and two may be easily integrated within a single housing with shared electronics. This also enables dual $\lambda_{1}$ and $\lambda_{2}$ observations to be combined at the sensor as a ratio to lessen data storage and transmission requirements.

Both $K_{d}\left(\lambda_{1}/\lambda_{2}\right)$ and ${\left[L_{W}\left(\lambda_{1}/\lambda_{2}\right)\right]}_{N}$ algorithmic approaches were shown to be suitable, because both had a MAD value less than 76%, but $K_{d}\left(320/780\right)$ was significantly superior overall (Table 3). In terms of the global domain, there are partitions wherein one algorithmic approach is nonetheless preferred or is an agreeable substitute. In oligotrophic waters the lower variance of the above-water $\lambda_{1}$ approach is likely suitable (Figure 5) when in-water sampling is challenging, e.g., water body access closed to prevent the spread of invasive species. In mesotrophic (typically coastal) waters, the variance for the ${\left[L_{W}\left(\lambda_{1}/\lambda_{2}\right)\right]}_{N}$ algorithm is more robust than ${\left[L_{W}\left(\lambda_{1}\right)\right]}_{N}$, indicating a need for two bands, whereas a $K_{d}\left(\lambda_{1}\right)$ algorithm can still suffice. For the latter, an ecosystem requiring diurnal study or with canopy effects, e.g., a kelp forest, is anticipated to require a hybridnamic in-water sensor Figure 6). Within increasingly shallow inland waters, turbidity, optical complexity, and eutrophic conditions predominate (although exceptions are possible, e.g., in lakes). When turbidity creates significant loss of signal, e.g., in the UV domain, a hybridnamic above-water approach is anticipated (Figure 7).

An examination of an algorithm to estimate $a_{\mathrm{CDOM}}\left(440\right)$ using $K_{d}\left(\mathrm{PAR}\right)$, i.e., $K_{d}$ based on PAR calculated from discrete spectral channels in near-surface waters (top 1 m) wherein no channels were flux limited, showed viability and deserves separate comment. The $K_{d}\left(\mathrm{PAR}\right)$ approach is potentially useful, because PAR has utility across multiple research disciplines and is the most widely deployed and recorded single-channel measurement in service, both on present and legacy AUV platforms. As such, these results show promise for the general applicability of a broadband PAR algorithm and are intended to encourage further investigation but should not be directly used without a careful consideration of the limitations inherent to single broadband detectors and the inherent impact of increasing depth on the spectral composition of downward irradiance in the water column.

## Figures and Tables

**Table 1:** The one-band $K_{d}\left(\lambda \right)$ algorithms, organized spectrally with individual λ values in units of nanometers, and based on linear and power functions as appropriate, i.e., y=mx+b and $y=ax^{k}$, respectively. The $N_{F}$ and $N_{V}$ variables usually differ from wavelength to wavelength, because of the random selection process used with the cross-validation technique (Section 2.4). The equivalents for the RMSD, MAD, and MBIAS terms expressed in percent are indicated in parentheses for all three parameters.

λ	Fitted Equation	$N_{F}$	$N_{V}$	$R^{2}$	RMSD	MAD	MBIAS
313	y=0.070x−0.001	202	59	0.986	0.063 (2.9%)	1.170 (17.0%)	0.997 (−0.3%)
320	y=0.079x−0.003	627	162	0.986	0.064 (3.0%)	1.154 (15.4%)	0.999 (−0.1%)
340	y=0.100x−0.002	645	144	0.976	0.066 (3.1%)	1.201 (20.1%)	0.999 (−0.1%)
380	$y\;=\;0.146x^{1.012}$	626	163	0.934	0.096 (4.5%)	1.351 (35.2%)	0.996 (−0.3%)
412	$y\;=\;0.187x^{1.038}$	667	122	0.884	0.134 (6.2%)	1.492 (49.4%)	0.996 (−0.3%)
PAR	$y\;=\;0.492x^{1.304}$	628	161	0.877	0.229 (10.7%)	1.536 (53.6%)	1.002 (−0.2%)

**Table 2:** The one-band ${\left[L_{W}\left(\lambda \right)\right]}_{N}$ algorithms following the formatting and reporting procedures established for Table 1, with the NOMAD results for ${\left[L_{W}\left(412\right)\right]}_{N}$ indicated as NOM. The NOMAD $a_{\mathrm{CDOM}}\left(440\right)$ data span approximately 0.001–1.116 m${}^{-1}$, compared to the C-OPS $a_{\mathrm{CDOM}}\left(440\right)$ range of 0.001–2.146 m${}^{-1}$.

λ	Fitted Equation	$N_{F}$	$N_{V}$	$R^{2}$	RMSD	MAD	MBIAS
313	$y\;=\;0.004x^{-1.210}$	233	51	0.876	0.311 (14.5%)	1.626 (62.6%)	1.001 (−0.1%)
320	$y\;=\;0.006x^{-1.043}$	638	151	0.851	0.499 (23.3%)	1.588 (58.8%)	1.002 (−0.2%)
340	$y\;=\;0.010x^{-1.167}$	625	164	0.825	0.795 (37.1%)	1.648 (64.8%)	1.004 (−0.4%)
380	$y\;=\;0.017x^{-1.277}$	625	164	0.747	0.495 (23.1%)	1.828 (82.8%)	1.005 (−0.5%)
412	$y\;=\;0.027x^{-1.497}$	635	154	0.648	0.362 (16.9%)	2.063 (106.3%)	1.007 (−0.7%)
NOM †	$y\;=\;0.031x^{-1.099}$	844	212	0.460	0.165 (14.8%)	2.165 (116.5%)	1.002 (−0.2%)

† NOMAD ${\left[L_{W}\left(412\right)\right]}_{N}$ data are provided as quality assured and were used unfiltered.

**Table 3:** The band-ratio $K_{d}\left(\lambda \right)$ and ${\left[L_{W}\left(\lambda \right)\right]}_{N}$ algorithms following the formatting and reporting procedures established for Table 1. To save table space, the ${}_{\lambda_{2}}^{\lambda_{1}}$ notation indicates the two bands in the ratio with $\lambda_{1}$ as the numerator term and $\lambda_{2}$ as the denominator term, i.e., Kdλ1λ2 is equivalent to $K_{d}\left(\lambda_{1}\right)/K_{d}\left(\lambda_{2}\right)$ and LWλ1λ2N equals ${\left[L_{W}\left(\lambda_{1}\right)\right]}_{N}/{\left[L_{W}\left(\lambda_{2}\right)\right]}_{N}$.

${}_{\lambda_{2}}^{\lambda_{1}}$	Fitted Equation	$N_{F}$	$N_{V}$	$R^{2}$	RMSD	MAD	MBIAS
$K_{d}\left({}_{780}^{320}\right)$	y=0.256x−0.003	630	159	0.996	0.037 (1.7%)	1.075 (7.5%)	0.999 (−0.1%)
$K_{d}\left({}_{670}^{412}\right)$	$y=0.165x^{1.268}$	636	153	0.916	0.146 (6.8%)	1.393 (39.3%)	0.997 (−0.3%)
$L_{W}\left[{}_{780}^{320}\right]{}_{N}$	$y=0.254x^{-0.544}$	629	160	0.913	0.140 (6.5%)	1.414 (41.4%) †	0.993 (−0.7%)
$L_{W}\left[{}_{670}^{412}\right]{}_{N}$	$y=0.232x^{-0.854}$	610	179	0.924	0.160 (7.4%)	1.398 (39.8%) †	0.998 (−0.2%)

† Although agreeing within the calibration uncertainty, differences are explained in the text.

## Data Availability

The data supporting the conclusions of this article will be made available by the lead author, without undue reservation.
